# Dental Splints in Dentoalveolar Trauma: An Overview of Public Healthcare Services in Brazil

**DOI:** 10.1111/edt.70057

**Published:** 2026-01-21

**Authors:** Caroline Garcia Orsi, Roberta Alves de Oliveira, Carlos Daniel Sturaro Junqueira, André Luis Faria‐e‐Silva, Liran Levin, Carlos José Soares, Priscilla Barbosa Ferreira Soares

**Affiliations:** ^1^ Department of Periodontology and Implantology, Faculty of Dentistry Federal University of Uberlândia – UFU Uberlândia MG Brazil; ^2^ Faculty of Dentistry Federal University of Uberlândia – UFU Uberlândia MG Brazil; ^3^ Department of Operative Dentistry, Faculty of Dentistry Université Laval Québec Quebec Canada; ^4^ College of Dentistry University of Saskatchewan Saskatoon Saskatchewan Canada; ^5^ Department of Operative Dentistry and Dental Materials, Faculty of Dentistry Federal University of Uberlândia – UFU Uberlândia MG Brazil

**Keywords:** avulsion, emergency treatment, fracture, luxation, tooth injuries

## Abstract

**Background/Aim:**

The prognosis of traumatized teeth might be influenced by the type and quality of the dental splint. This prospective observational study was aimed to evaluate the indications and quality of dental splints performed in a comprehensive public oral health service in the city of Uberlândia, Brazil.

**Materials and Methods:**

Between January 2024 and January 2025, 51 dental splints involving 320 teeth were evaluated. Data of the dental splint types (semi‐rigid or rigid), splint characteristics, polishing of resin composite, plaque retention, oral hygiene orientation, patient satisfaction, duration of the splint, and adherence to IADT protocol were recorded. Data were analyzed descriptively.

**Results:**

Most dental splints were semi‐rigid using fishing nylon line (58.8%); 49% showed improper alignment. Visible plaque was presented in 73.4% and gingival inflammation in 66.9% of the dental splints. Only 32.6% of treated patients received oral hygiene instructions. Diagnostic errors were found in 19.6% of cases, and 31.4% did not follow the recommended protocols regarding the correct dental splint rigidity.

**Conclusion:**

Gaps related to splint indication, application, and follow‐up were observed within the public healthcare services evaluated in this study. Reinforcing the necessity of training and continuing education, protocol standardization and better recording documentation are essential to enhance trauma care outcomes follow‐up.

**Trial Registration:** Brazilian Clinical Trials Registry (ReBEC) under the UTN code: U1111‐1309‐8338

## 1 Introduction

Traumatic dental injuries are common worldwide, affecting approximately 25% of school‐age children and 12.5% of the global population, with an estimated global incidence of 4.5% per year [1, 2, 3]. Effective clinical management of traumatic dental injuries requires prompt and structured interventions, including hard and soft tissues examination, radiographic detection of fractures, pulp sensibility testing, and appropriate dental splinting when indicated [4, 5, 6]. Dental splinting is a critical intervention after several dental trauma injuries, aiming to stabilize the repositioned teeth to promote optimal healing of the pulp, periodontal ligament, and alveolar bone [4, 6, 7, 8]. By stabilizing the traumatized teeth, dental splinting can maintain the correct position, favor tissue recovery, reduce the risk of complications such as root resorption or tooth loss, and improve patient comfort during the healing process [9].

An effective dental splint must provide stable fixation while permitting controlled physiological mobility to support periodontal healing [6, 7, 10, 11]. It must be positioned correctly avoiding the application of active forces that could interfere with the recovery process [4, 7]. The dental splint can also promote patient comfort, minimizing the negative impact on speech or daily activities [7, 12]. The application of the resin composite and adhesive system to stabilize the flexible or rigid components used for splinting fabrication should consider the gingival or proximal areas to minimize plaque accumulation and periodontal complications [4]. The removal of the splinting should be simple, atraumatic, and time‐efficient [7, 9, 13].

Diagnosis during the first consultation of dental trauma has notable challenges due to the complexity and variability of injury classification [1, 2, 14]. While most traumatic dental injuries are easily identifiable, the absence of standardized diagnostic protocols and limited examiner training in general public or private health services can compromise the consistent evaluations and emergency treatment, particularly in cases when multiple injuries are present [2, 14].

**FIGURE 1:**
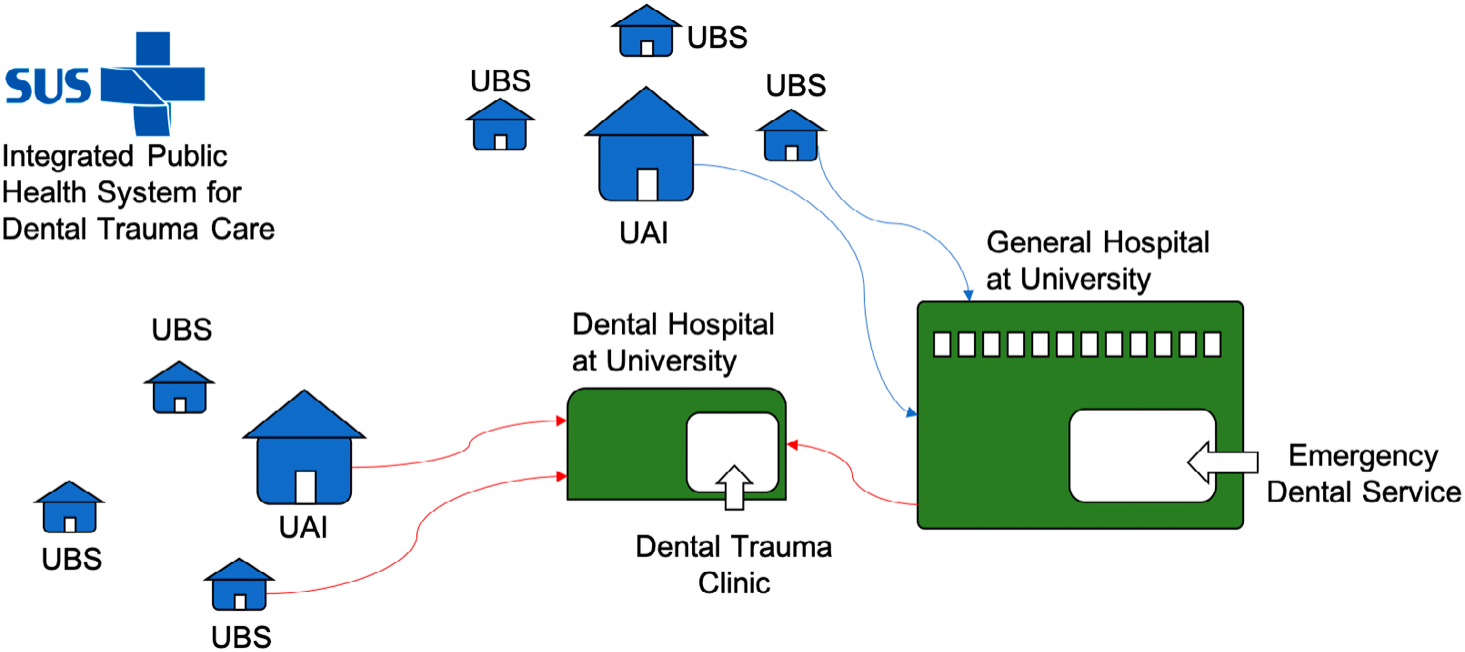
Schematic representation of the Brazilian public health system evaluated.

The Brazilian Unified Health System (SUS) plays a crucial role in ensuring access to quality healthcare in more than 5570 municipalities in Brazil [15], including dental trauma treatment. In Uberlândia (Figure 1), public oral health services are delivered through local Basic Health Units (UBS) and Integrated Care Units (UAI) that are strategically distributed around the regions of the municipality, with the objective to ensure equitable access to oral health services for the population [16]. The dental services in these units work during the daytime (7 a.m. to 5 p.m.). Due to the complexity of the emergency of dental trauma, patients are frequently referred by public health professionals to the Dental Emergency Service (PSO), located inside the General Hospital at the Federal University of Uberlândia, which serves as the official emergency dental reference unit for the municipality. Within this service, two care teams operate together, one composed of undergraduate students and the other by the Oral and Maxillofacial Surgery residents supervised by professors. The dental trauma clinical cases designated for each team depend on the complexity. The dental trauma emergency service is offered 24 h per day, during all days of the year. Tooth repositioning, pulp protection, soft tissue sutures, and provisional restorations are the most common dental trauma emergency treatments provided.

The Dental Trauma Clinic Federal University of Uberlândia is a specialized reference unit of the Public Unified Health System located at Dental Hospital, which provide free care for patients referred from the Dental Emergency Service (PSO), Primary Health Centers, and also from private services since 2003. Given the importance of the initial emergency response in dental trauma for ensuring a favorable long‐term prognosis, it is essential to evaluate whether the dental splints have been indicated and performed according to the type of dental trauma diagnosed. Additionally, it is important to assess whether their use complies with the basic principles of dental splinting recommended by the IADT guidelines to better design public policies for improving oral health care. Therefore, this study was aimed at conducting an internal assessment of the emergency dental care services of the Federal University of Uberlândia and the Primary Health Centers by evaluating the characteristics, diagnostic accuracy, clinical indication, and execution quality of dental splints performed and subsequently referred to the Dental Trauma Clinic, to identify deficiencies and propose strategies for improving clinical practices.

## 2 Materials and Methods

A clinical, observational, prospective study was conducted at the Dentoalveolar Trauma Clinic of the Federal University of Uberlândia, Brazil, between January 2024 and January 2025 in accordance with PROBE 2023 guidelines [17]. The study protocol was approved by the Ethics Committee of the Federal University of Uberlândia (CAAE: 77922324.6.0000.5152) and registered in the Brazilian Registry of Clinical Trials (ReBEC UTN‐code: U1111‐1309‐8338).

### 2.1 Sample and Patient Selection

A convenience sample was used [18], including all patients referred to the Dental Trauma Clinic at the Dental Hospital of the Federal University of Uberlândia who had at least one traumatized permanent tooth stabilized by dental splint. Patients were referred from the Primary Health Centers, Oral and Maxillofacial Surgery Service, or the Dental Emergency Service. All patients provided written informed consent in accordance with the Declaration of Helsinki.

### 2.2 Clinical, Radiographic and Photographic Evaluation

At the first examination, a clinical evaluation was performed. Periapical radiographs and intraoral photographs were obtained to diagnose and record the dental trauma.

### 2.3 Splint Evaluation Protocol

An evaluation form was completed for each case to record: the referring unit where the local of the patient was attended, type of splint used (rigid or semi‐rigid), material used for dental splint including 0.9 and 0.6 mm stainless steel wire (Morelli Ortodontia, Sorocaba, SP, Brazil), 1.0‐mm fishing nylon line (Mazzaferro, Diadema, SP, Brazil), and saline plastic strip obtained from saline solution packaging (Fresenius Kabi, Aquiraz, CE, Brazil), the splint extension (the number of the teeth included), dentition type (permanent or mixed), splint positioning (middle, cervical, or incisal coronal third), alignment (adequate or inadequate, considered inadequate if the splint was not positioned at the middle third or its extension did not include at least the adjacent teeth), oral hygiene condition (adequate or inadequate), presence and type of periodontal injuries (concussion, subluxation, lateral luxation, intrusion, extrusion and avulsion), dental injuries (enamel crack, enamel fracture, enamel and dentin fracture, enamel, dentin and pulp fracture, crown‐root fracture, and root fracture) and bone injuries (alveolar wall fracture, alveolar bone fracture–vestibular or lingual/palatal).

### 2.4 Tooth‐Level Evaluation

**FIGURE 2:**
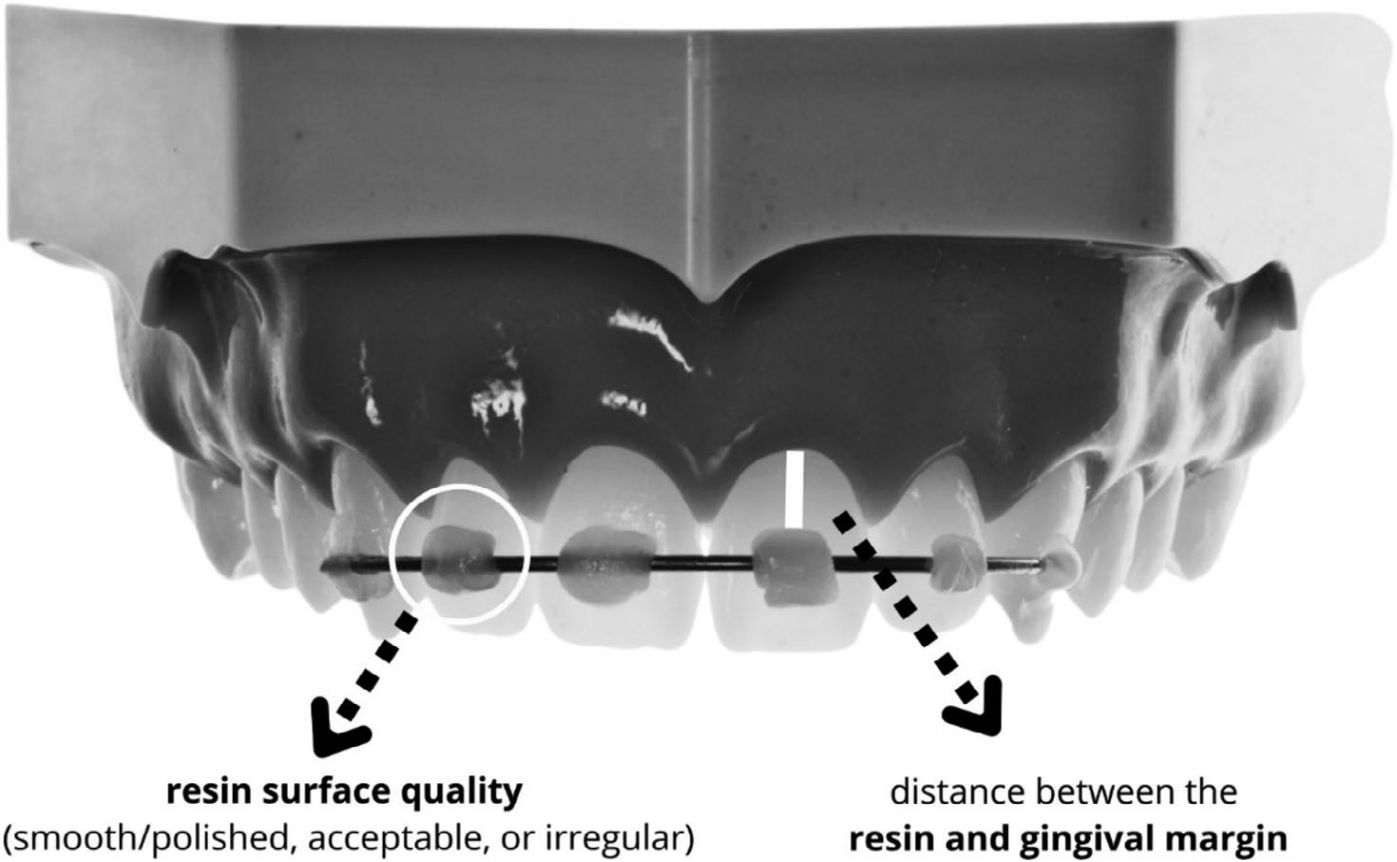
Schematic representation of the evaluation of teeth involved in splints.

All involved teeth in the dental splints were assessed regarding the splint retention (whether resin composite or wire were detached), distance between the resin composite and gingival margin (Figure 2), resin composite surface polishing (smooth/polished, acceptable, or irregular), presence of visible plaque, and the presence of gingival inflammation. A millimeter‐marked North Carolina No. 15 periodontal probe with a single tip (Hu‐Friedy, Chicago, IL, USA) was used, and only one operator performed all the probing measurements.

### 2.5 Patient Satisfaction and Information Assessment

**FIGURE 3:**
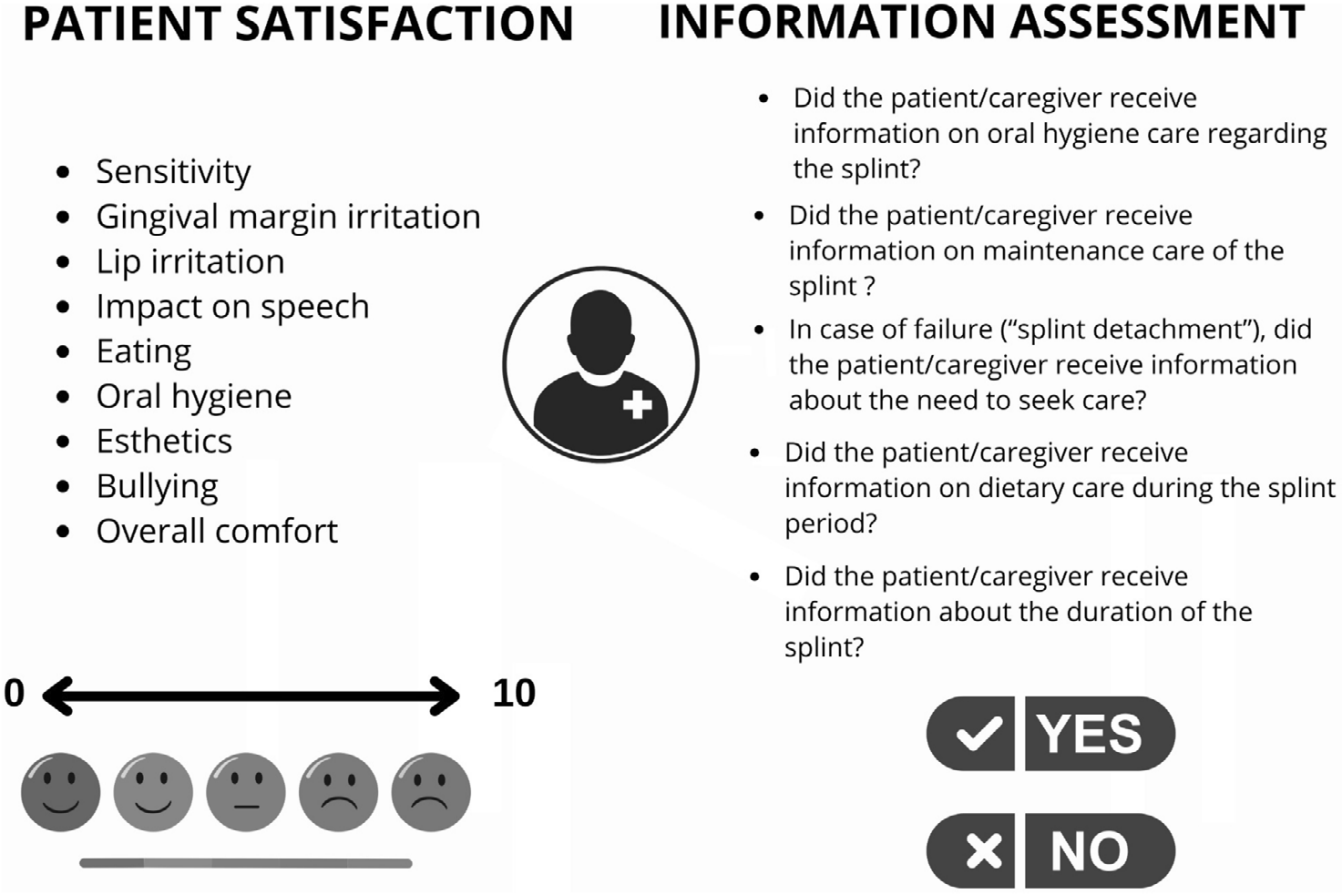
Schematic representation of the information and patient satisfaction assessment.

Patient satisfaction was assessed using an 11‐point Likert scale ranging from 0 to 10 [19], covering the following domains: sensitivity, gingival margin irritation, lip irritation, impact on speech, eating, oral hygiene, esthetics, bullying, and overall comfort [20]. Additionally, the information provided during the emergency visit at the referring unit was evaluated. This included whether patients received guidance regarding oral hygiene, diet, care in case of splint detachment, maintenance, and estimated splint duration (Figure 3).

### 2.6 Evaluation of Treatment Adequacy

The diagnostic errors of the diagnoses established at the referring unit were checked at the Dental Trauma Clinic. The splint indication according to the International Association of Dental Traumatology (IADT) guidelines, which recommend rigid splints in cases involving bone fracture, was also evaluated [21]. The total splinting time until splint removal was recorded.

### 2.7 Referral Form Assessment

The referral forms were analyzed to determine whether they recorded information regarding the splint type, material used, splint extension, trauma history, and type of trauma.

### 2.8 Treatment Planning and Execution

Based on the diagnostic assessment at the Dental Trauma Clinic, individualized treatment plans were developed. All treatments were carried out by undergraduate students enrolled in the last semester of the Dental School curriculum at Federal University of Uberlândia. Treatments were conducted under faculty supervision, strictly following the personalized plans and clinical protocols, with appropriate monitoring and follow‐up, following the IADT guidelines.

### 2.9 Statistical Analysis

Statistical analysis was conducted using Jamovi software (version 2.6; The jamovi project, 2024), operating on the R statistical computing platform (version 4.4; R Core Team 2024). The analysis was descriptive, aiming to summarize the characteristics of the clinical aspects of the splints evaluated. All variables were categorical and reported as absolute frequencies (*n*) and percentages (%).

## 3 Results

In total, 49 patients, 51 splints and 320 teeth involved in the splints were evaluated. Dental, periodontal, and bone injuries according to tooth type and injury category are described in Table 1. A total of 86 periodontal injuries, 84 tooth injuries, and 58 bone injuries were detected. Among periodontal injuries, the maxillary central incisor accounted for 64.0% of cases and the most common types were avulsion (26.7%). The maxillary central incisor was involved in 50.0% of the tooth injuries. Enamel fracture was the most prevalent type (44.0%). The maxillary central incisor was the most affected (44.8%) with bone injuries, with a predominance of alveolar wall fracture (37.9%).

**TABLE 1:** Distribution of dental, periodontal ligament, and bone injuries according to tooth type and injury classification.

	*N*	% of Total
*Tooth involved with periodontal injury*		
Maxillary central incisor	55	64.0
Maxillary lateral incisor	20	23.2
Mandibular central incisor	7	8.1
Mandibular lateral incisor	3	3.5
Maxillary canine	1	1.2
*Type of periodontal injury*		
Avulsion	23	26.7
Lateral luxation	22	25.6
Subluxation	20	23.3
Extrusion	18	20.9
Intrusion	2	2.3
Concussion	1	1.2
*Tooth involved with dental injury*		
Maxillary central incisor	42	50.0
Maxillary lateral incisor	21	25.0
Maxillary canine	7	8.3
Mandibular central incisor	4	4.8
Mandibular lateral incisor	4	4.8
Mandibular canine	3	3.6
Others	3	3.6
*Type of tooth injury*		
Enamel fracture	37	44.0
Enamel/dentin fracture	28	33.3
Apical root fracture	7	8.3
Enamel/dentin/pulp fracture	6	7.1
Enamel crack	4	4.8
Crown‐root fracture	2	2.4
*Tooth affected with bone injury*		
Maxillary central incisor	26	44.8
Mandibular central incisor	10	17.2
Maxillary lateral incisor	7	12.1
Mandibular lateral incisor	6	10.3
Others	4	6.9
Mandibular canine	3	5.2
Maxillary canine	2	3.4
*Type of bone injury*		
Alveolar wall fracture	22	37.9
Alveolar bone fracture (V)	19	32.8
Alveolar bone fracture (L/P)	17	29.3

**FIGURE 4:**
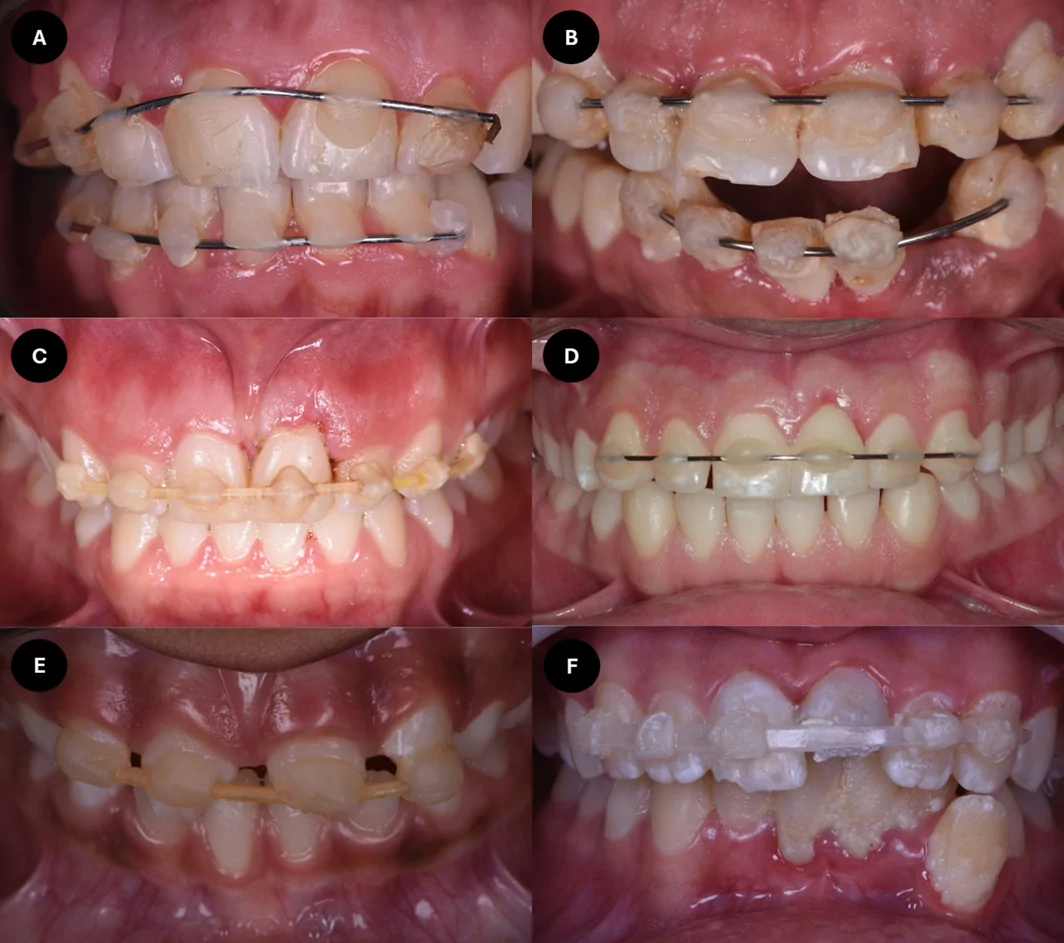
Clinical examples of different dental splints performed after dentoalveolar trauma in the public healthcare unit: (A) Rigid splint using 0.9 mm stainless steel wire, with inadequate alignment and cervical positioning. (B) Rigid splint with 0.9 mm stainless steel wire, showing resin composite excess, poor polishing, and located at cervical positioning. (C) Semi‐rigid splint using fishing nylon line, with acceptable positioning, but with visible plaque accumulation. (D) Rigid splint using 0.6 mm stainless steel wire, properly aligned at the middle third, with a smooth and polished resin composite surface. (E) Semi‐rigid splint with fishing nylon line, presenting an irregular resin composite surface and plaque retention. (F) Splint made with a saline plastic strip, showing irregular surface and visible plaque accumulation.

The characteristics assessed of the evaluated dental splints are presented in Table 2. Most splints were performed at the Emergency Center by Oral and Maxillofacial residents (60.8%), and semi‐rigid splints were more frequently used (58.8%). The most used material was 1.0 mm thick fishing nylon line (51.0%), followed by 0.9 mm stainless steel wire (41.2%). Regarding splint extension, 29.4% involved the upper canine to canine teeth. The majority of splints were positioned mainly at the middle third of the crown (56.9%); splint alignment was considered adequate in 51.0% of cases. However, more than half of the patients (54.9%) presented inadequate oral hygiene. Representative clinical examples of rigid and semi‐rigid splints, using different materials, positioning, and surface quality, are shown in Figure 4A–F.

**TABLE 2:** Distribution of splint characteristics and clinical parameters.

	*N*	%
*Emergency care location*		
Oral and maxillofacial surgery service	31	60.8
Dental emergency center	14	27.4
Primary health centers	6	11.8
*Rigidity of the splint*		
Semi‐rigid	30	58.8
Rigid	21	41.2
*Material used for splint production*		
1 mm thick fishing nylon line	26	51.0
0.9 mm stainless steel wire	21	41.2
Saline solution	3	5.9
0.6 mm stainless steel wire	1	2.0
*Extension of the splint*		
Others	21	43.2
Upper canine to canine	15	29.4
Upper first premolar to first premolar	9	17.6
Lower canine to canine	5	9.8
*Type of dentition*		
Permanent	44	86.3
Mixed	7	13.7
*Dental coronal position of the splint*		
Middle third (contact point)	29	56.9
Cervical third	18	35.3
Incisal third	4	7.8
*Splint alignment*		
Adequate	26	51.0
Inadequate	25	49.0
Oral hygiene	N	%
Inadequate	28	54.9
Adequate	23	45.1

The tooth‐by‐tooth evaluation of the splints is described in Table 3. A total of 320 teeth were evaluated. Detachment of the splint material was observed in only 3.1% of cases. Most resin composite margins were positioned more than 3 mm from the gingival margin (69.1%), while 13.1% were in direct contact with the gingival tissue (0 mm). In terms of polishing, 49.7% of splints had acceptable surface quality. Visible plaque was detected in 73.4% of the teeth, and gingival inflammation was present in 66.9% of cases.

**TABLE 3:** Frequency distribution of clinical parameters of the splint per tooth.

	*N*	%
*Splint retention*		
Stable	310	96.9
Detached	10	3.1
*Distance resin composite to gingival margin* (mm)		
1‐3	217	69.1
> 3	56	17.8
0	41	13.1
*Polishing score*		
Acceptable (2)	156	49.7
Smooth (1)	113	36.0
Irregular (3)	45	14.3
*Visible plaque presence*		
Present	235	73.4
Absent	85	26.6
*Gingival inflammation*		
Present	214	66.9
Absent	106	33.1

The clinical parameters per splint are described in Table 4. In general, each splint involved 6.3 teeth, with a detachment rate of 3.0%. The mean distance from the resin to the gingival margin was 2.2 mm, and the mean polishing score was 1.8, indicating a tendency toward acceptable surface quality. Additionally, 72.2% of teeth presented visible plaque and 64.6% showed signs of gingival inflammation.

**TABLE 4:** Clinical parameters of the splinted teeth.

Clinical parameters of splinted teeth	Mean (SD)
Number of teeth per splint (*n*)	6.3 (1.4)
Teeth with detachment (%)	3.0 (7.7)
Distance from resin to gingival margin (mm)	2.2 (1.2)
Polishing score (1–3)	1.8 (0.5)
Teeth with visible plaque (%)	72.2 (39.4)
Teeth with gingival inflammation (%)	64.6 (40.2)

The patient satisfaction regarding clinical outcomes is described in Table 5. The highest satisfaction scores were related to oral hygiene (5.4), aesthetics (5.3), and eating (4.9). Lower levels of discomfort were reported for gingival margin irritation (2.1) and lip irritation (2.5). Speech impairment and tooth sensitivity had intermediate scores of 3.2 and 3.9, respectively. Notably, bullying had the lowest score (0.6), indicating minimal reported impact. Overall comfort was rated at 4.4.

**TABLE 5:** Patient satisfaction regarding various clinical outcomes.

Patient satisfaction	Mean (SD)
Tooth sensitivity	3.9 (3.4)
Gingival margin irritation	2.1 (3.1)
Lip irritation	2.5 (3.5)
Impact on speech	3.2 (3.5)
Impact for Eating	4.9 (3.6)
Oral hygiene	5.4 (3.3)
Esthetics	5.3 (4.4)
Bullying	0.6 (1.8)
Comfort	4.4 (3.8)

The frequency of guidance provided to the patients on post‐splinting care was described in Table 6. Of the 49 patients involved in this study, 18.4% did not respond to this section of the questionnaire. Among the 40 patients who did, the most frequently addressed topic was diet (65.3%).

**TABLE 6:** Frequency distribution of guidance provided on key aspects of post‐splinting care.

Guidance provided for patients	Yes (%)	No (%)	Not specified (%)
Oral hygiene	16 (32.6)	24 (49.0)	9 (18.4)
Retention	12 (24.5)	28 (57.1)	9 (18.4)
What to do in case of detachment	8 (16.3)	32 (65.3)	9 (18.4)
Diet	32 (65.3)	8 (16.3)	9 (18.4)
Information on duration of the splint	16 (32.6)	24 (49.0)	9 (18.4)

Most splints complied with the IADT protocol (35/51; 68.6%). Among the 16 non‐compliant cases, 87.5% were made on Emergency Service, being 56.3% made by Oral and Maxillofacial Residents and 31.2% made by undergraduate students, and 12.5% were made on Primary Health Centers. The splints that were non‐compliant with the IADT guideline, 29.0%, were made by Oral and Maxillofacial Surgery residents, 35.7% by the undergraduate students in Dental Emergency Service at General Hospital, and 33.3% made by clinicians in the Primary Health Centers at the municipality. Additionally, diagnostic errors were identified in 19.6% of cases (*n* = 10).

**FIGURE 5:**
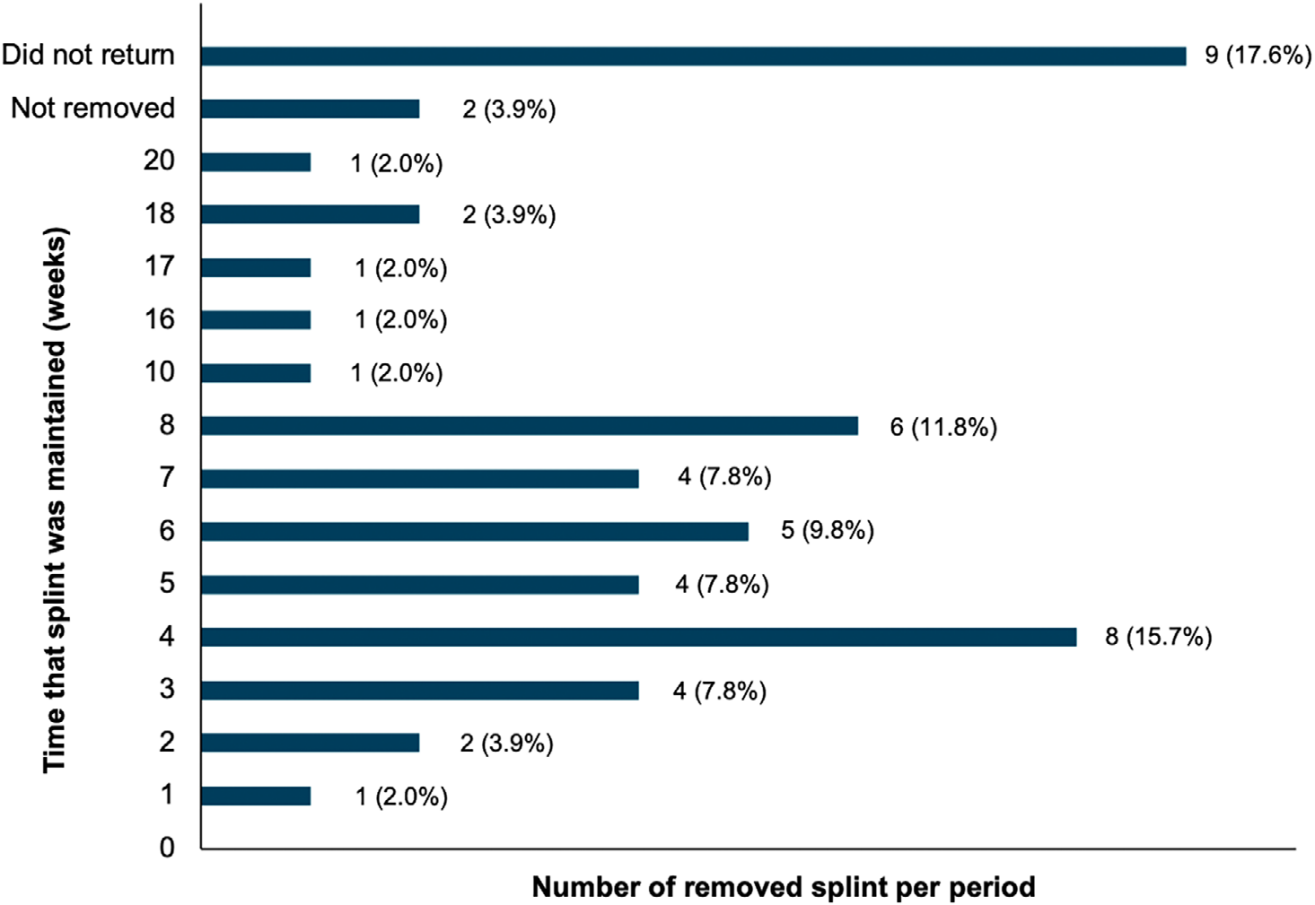
Distribution of splint removal per period.

**FIGURE 6:**
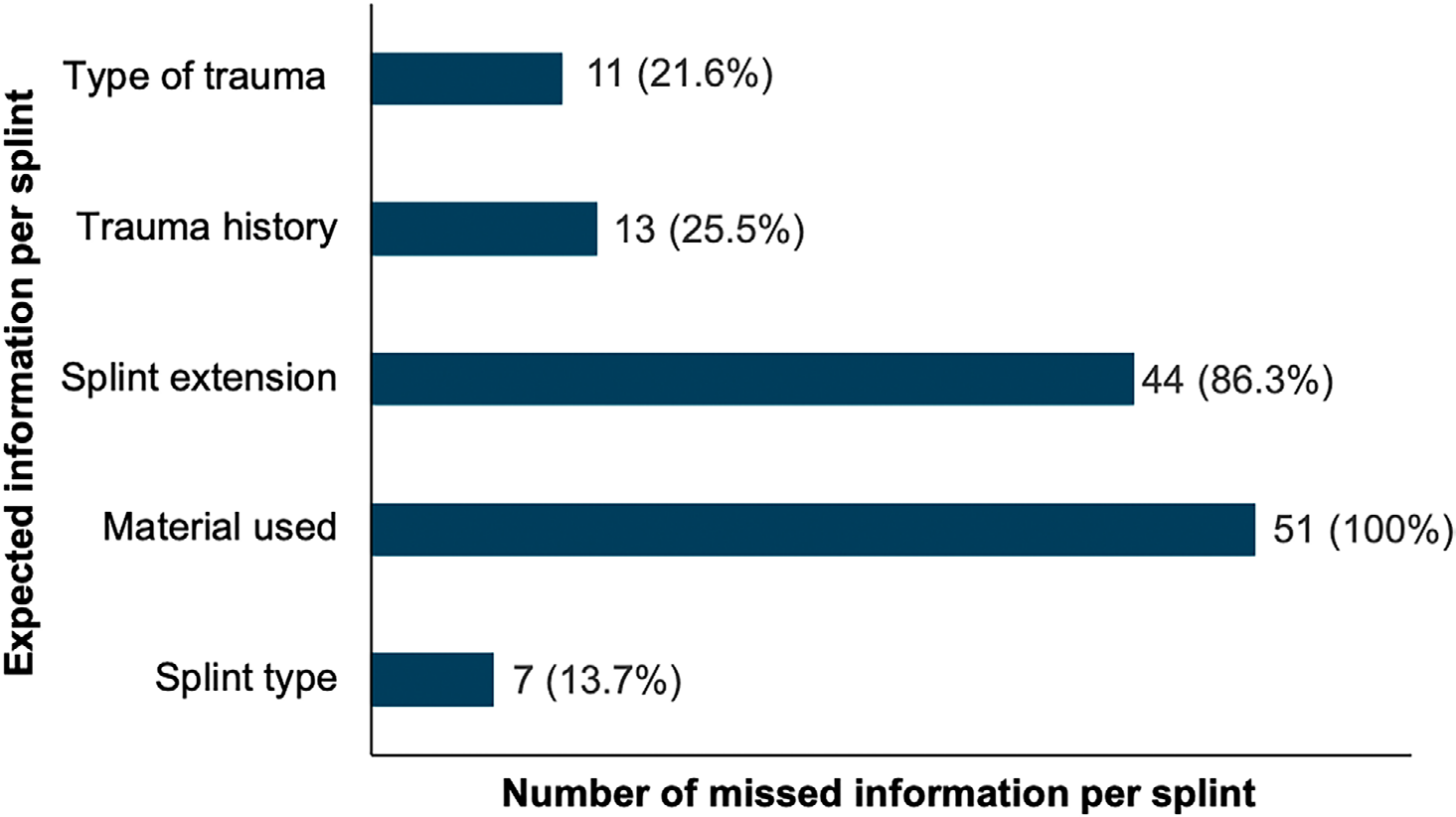
Frequency distribution of splint with missing information in referral forms.

The distribution of splint removal was described in Figure 5. Most splints were removed after 3–8 weeks, with the highest frequency at 4 weeks. The frequency of missing information in referral forms was described in Figure 6. The splint material was not reported in any of the cases (100.0%), while the type of trauma experienced was missing in 21.6% of the forms.

## 4 Discussion

This study investigated the diagnostic accuracy, indication criteria, and technical execution of dental splints applied across different healthcare units and subsequently referred to the Dental Trauma Clinic of the Federal University of Uberlândia. A predominance of semi‐rigid splints using fishing nylon line and rigid splints using 0.9 mm stainless steel wire was noted. High prevalence of periodontal inflammation and significant variability in splint positioning was observed. Most splints adhered to IADT guidelines. However, discrepancies in technique, timing, and communication between clinicians and patients were evident. These findings emerged within the context of the Brazilian Unified Health System (SUS), which provides universal and free access to emergency and specialized dental care through public emergency units and structured referral pathways across municipalities, while operating under high patient demand, limited clinical time, and heterogeneous resources. Additionally, uneven training in dental traumatology across dental schools and public services may compromise diagnostic accuracy, technical consistency, and adherence to international guidelines in routine practice. Regarding trauma types, the most commonly affected teeth were the maxillary central incisors (64.0%) [5]. Subluxation, avulsion, and lateral luxation were the predominant injury types, all of which require careful stabilization to ensure periodontal healing and pulp vitality [22]. Lateral luxation and avulsion, particularly, pose high risks for complications like root resorption and ankylosis if splinting is not properly indicated [22, 23]. Providing quick, evidence‐based care that considers both the type of injury and the location of the affected tooth is key to maintaining long‐term function and esthetics.

Analysis of splint rigidity and material selection revealed adherence to best practices: rigid splints predominantly utilized the appropriate 0.9 mm orthodontic stainless‐steel wire (41.2%) [21], while fishing nylon line (51.0%) was most used for semi‐rigid splints, both are in accordance with current guidelines [4, 6, 7, 11]. In addition, most splints were positioned at the middle third (56.9%), corresponding to the contact point, which is consistent with the basic principles of splinting and contributes to effective tooth stabilization and proper oral hygiene [4, 12]. However, a significant number of the splints were placed on the cervical third (35.3%) or exhibited inadequate extension (43.2%), often failing to include adjacent teeth as required for optimal stabilization [11]. This limitation frequently resulted in inadequate alignment (49.0%). The misalignment of the splint can generate the force inducing movement of the traumatized teeth (Figure 4A). These issues can reduce splint effectiveness and compromise the long‐term prognosis. A high prevalence of visible plaque (73.3%) and gingival inflammation (66.9%) reflects compromised periodontal health among splinted teeth, a pattern also observed at the splint level, where the average percentage of teeth with visible plaque and gingival inflammation per splint was 72.2% and 64.6%, respectively. Additionally, oral hygiene was classified as inadequate in 54.9% of the cases. The mean distance from resin composite to the gingival margin was 2.2 mm, but in 13.1% of cases, the resin was in direct contact with the gingival margin, and more 35.5% of splints were positioned at the cervical third, facilitating the biofilm accumulation [24, 25]. Similar periodontal implications of splint design and positioning have been reported in a Swiss clinical study, in which resin‐based trauma splints were associated with increased gingival irritation due to impaired access for oral hygiene [20]. Irregular resin composite surface was observed in 14.3% of the cases. Rougher resin composite surface might contribute to the presence of inflammation due to biofilm adhesion [26]. Moreover, 49.0% of patients reported not receiving hygiene instructions after splint placement. These findings reinforce that not only the technical aspects of splint fabrication and positioning, but also the communication between clinicians and patients, can minimize biofilm accumulation and protect periodontal tissues during stabilization [27].

Patient satisfaction was generally moderate to high, with higher ratings particularly evident in esthetics, oral hygiene, and overall comfort domains. The notably low scores reported for experiences related to bullying indicate that the visual aspects of the splints were largely acceptable and minimally intrusive. This outcome is particularly important in pediatric and adolescent populations, where the esthetic impact of dental interventions can significantly influence self‐esteem, social interactions, and overall psychological well‐being [28]. The results reinforce the clinical importance of selecting the materials and bonding procedures designed to minimize visibility and psychological distress [7]. However, moderate dissatisfaction was observed in areas associated with eating and speech impact, specifically functional limitations related to the splint design. Although this study had no aim to investigate the underlying reasons for this functional discomfort, splints with increased thickness, insufficient polishing, or rigid materials can limit tongue mobility, compromise speech clarity, and negatively affect mastication efficiency [20].

Regarding the information provided at the referral forms from the first location that attended the patients, it is concerning that 49.0% of patients had not received any guidance on oral hygiene orientation and regarding the expected duration of splint use. This lack of instruction can significantly compromise treatment outcomes, as inadequate hygiene around the splinted area increases the risk of plaque accumulation, gingival inflammation, and periodontal complications [25]. Furthermore, not knowing the expected time frame for splint use may affect patient adherence to follow‐up visits and delay the removal of the device, potentially leading to unnecessary complications [29]. Additionally, 57.1% of patients reported receiving no information on how to maintain the retention of the splint, which can result in unintentional manipulation or damage to the appliance [30]. Most notably, 66.3% were not informed about what to do if the splint became detached, a situation that may lead to prolonged instability of the traumatized teeth, delayed healing, or even treatment failure if the patient does not seek timely professional assistance [23, 31, 32].

This study identified several instances (31.4%) in which the type of splint used was not consistent with the protocols established by the International Association of Dental Traumatology (IADT). According to these guidelines, rigid splints should be reserved for cases involving alveolar bone fractures, while flexible or semi‐rigid splints are indicated for periodontal ligament injuries [4, 21]. In this sample, however, some cases of ligament injuries were managed with rigid splints, and conversely, cases of alveolar fractures were stabilized using semi‐rigid devices. An Australian IADT‐based survey demonstrated heterogeneity in splint rigidity selection across luxation‐related clinical scenarios, including those with associated alveolar fractures [33]. These inconsistencies point to internal deficiencies in clinical decision‐making, even within the patients attended on Dental Emergency Service at the General Hospital. Implementing injury‐specific diagnostic checklists, step‐by‐step splint selection flowcharts based on IADT recommendations, and mandatory supervision of splint placement by trained staff during emergency care could help reduce such discrepancies. Proper recognition of injury type and selection of the appropriate splinting method are critical for favorable outcomes and long‐term tooth survival [23, 34].

Approximately 49.0% of cases involved splints that remained in place for more than 4 weeks. It is important to clarify that many of these cases were initially managed by the Oral and Maxillofacial residents on the Dental Emergency Service at the General Hospital. It is due to the severity of the trauma, often systemic in nature, requiring hospitalization and close medical monitoring. In such scenarios, immediate dental follow‐up may not be a clinical priority, and prolonged splinting becomes an unavoidable consequence of the patient's overall recovery trajectory. A hospital‐based audit conducted in the United Kingdom demonstrated that delayed splint removal was frequently associated with emergency‐led care pathways and challenges in follow‐up [35]. Therefore, the extended immobilization in these cases might not reflect a clinical error, but rather the complexity of interdisciplinary trauma care and the need to stabilize patients holistically before referral to specialized dental services [36].

An issue encountered in this study was the limited quality of documentation in referral forms. Many arrived with insufficient clinical details, lacking information on the type of injury, trauma chronology, or even the splinting technique used. This compromised the trauma clinic team to validate or adapt prior interventions. This also resulted in redundant imaging or delays in re‐planning. Inadequate records and rushed or poorly trained initial assessments frequently lead to errors in diagnosis, inappropriate splinting, and missed opportunities for optimal management [29]. In trauma cases, where diagnosis and timing are critical, poor communication and a lack of standardized documentation between services can directly impact clinical decisions and patient outcomes [29, 37]. Beyond being an operational issue, this highlights a need for better training regarding documentation during emergency care, irrespective if it is located at the University or at the municipality.

The mismanagement of complex injuries along with frequent errors in splint indication and technique suggests persistent gaps in trauma education and training, even at the postgraduate level [38, 39]. Scarce exposure to acute trauma cases, limited pre‐clinical simulation and insufficient structured assessment may hinder the consistent application of IADT recommendations in clinical practice, helping to explain the variability in splint indication, execution, and follow‐up observed in the present study [40, 41]. Furthermore, evidence suggests that professionals with greater clinical experience in trauma care tend to perform better diagnostically and therapeutically [33, 36], while poor judgment in the selection and duration of splinting can negatively affect healing and outcomes [39]. Thus, rather than an external audit, this study serves as a self‐assessment of institutional practices, aiming to identify weaknesses, promote targeted improvements in teaching and clinical protocols, and ultimately enhance the quality of care provided within the integrated public health system for treating dental trauma [42]. Although the study's observational design limits causal interpretations, and the heterogeneity among referring units may have influenced the choice and quality of splinting, the clinical insights it provided are highly relevant. The lack of long‐term follow‐up data also prevented us from evaluating outcomes like pulp vitality, root resorption, or long‐term tooth survival, reinforcing that any prognostic interpretation remains preliminary. Still, by analyzing real cases within the public health system, the study brings to light everyday challenges that often go unnoticed in controlled research settings. We observed inconsistencies in splint indication, diagnostic errors regarding the type of trauma, mistakes in the selection and execution of splinting techniques, delays in referral that extended splint duration, and missed follow‐up appointments that impacted treatment timelines. These are not isolated incidents; they reflect gaps in protocol standardization, interprofessional communication, and patient management. Alongside these limitations, positive aspects were also identified, including the widespread use of semi‐rigid splints aligned with international recommendations and moderate to high levels of patient satisfaction, particularly regarding esthetics and overall comfort, indicating that clinicians are able to apply guideline‐based approaches even in high‐demand public settings. Taken together, this balance between strengths and limitations highlights the influence of professional training, clinical protocols, and system‐level organization on the quality of dentoalveolar trauma care. The present study identified the need for structural improvements in both clinical and educational processes and then proposes a series of targeted interventions that could be applied broadly. These include developing theoretical modules for continuing education of dental professionals, online training with assessment tools for students, and the inclusion of dentoalveolar trauma content in residency curriculum, with emphasis on hands‐on training in splint fabrication. Standardized referral forms with essential fields can optimize clinical documentation and improve communication between services. Technological innovations may also play an important role worldwide, such as artificial intelligence systems capable of supporting the identification of dentoalveolar trauma cases and mobile applications providing diagnostic and treatment protocols for professionals, along with post‐trauma guidance for patients. Although not yet implemented, these strategies offer a roadmap for strengthening the quality and coordination of dental trauma care across healthcare systems [40, 41].

## 5 Conclusions

Although the evaluated integrated public service is comprehensive and provides free dental trauma treatment for the population, variability in the diagnosis of the type of trauma injury, indication, and execution of dental splints was found across different units of the evaluated Brazilian Unified Health System. Despite adherence to the IADT protocols, in many cases, diagnostic errors, improper splint positioning, lack of hygiene instructions, and prolonged retention periods were observed. These findings reveal limitations in documentation, necessity for professional training and awareness for correct follow‐up. Standardized forms and continued education in dental trauma care are essential to improve the consistency and quality of treatment provided by the evaluated public service. Furthermore, larger‐scale studies with structured long‐term follow‐up are necessary to strengthen prognostic understanding and support future improvements.

## Author Contributions

C.G.O. investigation, methodology, formal analysis, writing – original draft. R.A.O. investigation, methodology. C.D.S.J. investigation, methodology. A.L.F.‐S. data curation, formal analysis, writing, review and editing. L.L. conceptualization, writing – review and editing. C.J.S. conceptualization, data curation, formal analysis, writing – review and editing. P.B.F.S. supervision, conceptualization, funding acquisition, project administration, resources, writing, review and editing. All authors gave final approval of the version to be published.

## Funding

This work was supported by Coordenação de Aperfeiçoamento de Pessoal de Nível Superior, Finance code 001. Conselho Nacional de Desenvolvimento Científico e Tecnológico, INCT‐Saúde Oral e Odontologia, CNPq—Grants 406840/2022‐9, 422603/2021‐0. Fundação de Amparo à Pesquisa do Estado de Minas Gerais, APQ‐04262‐2, RED‐00204‐23.

## Ethics Statement

This study was approved by the Human Research Ethics Committee of the Federal University of Uberlândia (CAAE: 77922324.6.0000.5152).

## Consent

All the participants signed informed consent.

## Conflicts of Interest

The authors declare no conflicts of interest.

## Data Availability

The data that support the findings of this study are available on request from the corresponding author. The data are not publicly available due to privacy or ethical restrictions.
